# MAE-YOLO improves small object detection for intelligent inspection

**DOI:** 10.1038/s41598-026-57661-5

**Published:** 2026-06-13

**Authors:** Zhixian Chen, Yi Wang, Yuxing Bai, Ruidong An, Meiting Zhu, Wen Zhang, Jianwei Zhang

**Affiliations:** 1 Department of Computer Science and Technology, Taiyuan University, Taiyuan, 030032 China; 2 Beijing Reliable Agent Technology Co., Ltd., Taiyuan, 030032 China; 3 Technical Aspects of Multimodal Systems (TAMS), University of Hamburg, Vogt-Köln-StraBe 30, 22537 Hamburg, Germany

**Keywords:** Computer science, Information technology, Software, Scientific data, Electrical and electronic engineering

## Abstract

Intelligent inspection technology has become increasingly popular in industrial fields such as power facility maintenance (e.g., identifying cracked insulators, corroded transformers), traffic management (e.g., detecting vehicle anomalies), and industrial equipment upkeep (e.g., spotting surface defects on machinery, verifying the presence of small components like bolts and valves). The targets in these scenarios are often small-sized, well-defined objects that are critical to operational safety and efficiency. Although the traditional deep learning methods such as YOLO series have made progress in this field, however, in the detection of small targets under complex background, it still suffers from high false detection and miss rates, as well as high computational complexity, making it difficult to meet the real-time requirements of practical applications. In this article, an intelligent inspection model named MAE-YOLO is proposed. Firstly, a multi-scale edge space feature extraction module is proposed to optimize the edge and space feature extraction, which significantly improves the detection accuracy of small targets. Meanwhile, the adaptive multi-scale context fusion network is introduced to integrate the features of different scales effectively, thereby enhancing the robustness and adaptability of the model in dynamic environments. Finally, we propose an adaptive cavity shared detection head to further reduce the false detection and missing detection rate in multi-scale detection. By synergistically integrating MSESTE for edge-aware feature extraction, AMCFN for adaptive multi-scale context fusion, and ELCIN for parameter-efficient detection, MAE-YOLO achieves both lightweight design and high accuracy for small objects. Experimental results on the VisDrone2019 dataset show that the accuracy of MAE-YOLO is improved by 2.6% compared to the original YOLOv8n model. According to the results of the self-collected dataset, it can be seen that MAE-YOLO only needs 4.7 MB, which is reduced by 24% compared with YOLOv8n, while maintaining high detection accuracy. Unlike existing lightweight methods that often sacrifice edge details for efficiency, MAE-YOLO preserves fine-grained object boundaries through Sobel-based edge enhancement while reducing detection head parameters by 53%, achieving a superior accuracy-size trade-off (35.1% mAP@50 at 4.57 MB) compared to recent detectors such as SOD-YOLO (30.08% mAP@50). To facilitate reproducibility and further research, the source code of MAE-YOLO has been released at: https://github.com/970334745/MAE-YOLO.

## Introduction

In recent decades, with the rapid development of human–computer interaction, intelligent inspection technology has become increasingly popular in industrial fields such as power facility maintenance (e.g., identifying cracked insulators, corroded transformers), traffic management (e.g., detecting vehicle anomalies), and industrial equipment upkeep (e.g., spotting surface defects on machinery, verifying the presence of small components like bolts and valves). The targets in these scenarios are often small-sized, well-defined objects that are critical to operational safety and efficiency. The traditional manual inspection mode is limited by low detection efficiency, high cost and significant safety risks, especially in hazardous or complex operational environments where these limitations become particularly pronounced. Therefore, leveraging computer vision and artificial intelligence to automate the inspection process has become a key technical approach to improving inspection efficiency and detection accuracy^1–3^. Quadruped inspection robots, with their exceptional stability and flexibility, are capable of adapting to various complex terrains, including underground pipelines and the interiors of nuclear power plants, making them an ideal choice for industrial inspection and security monitoring^4–7^. However, these robots are inherently constrained by limited onboard computing power, strict real-time processing demands, and energy efficiency requirements. These operational constraints directly guided our design choices. First, the real-time processing requirement demands extremely low inference latency; therefore, we designed the Efficient Layer-Shared Convolution with Independent Normalization (ELCIN) head, which shares convolutional kernels across detection scales to drastically reduce computational cost while maintaining high accuracy. Second, the limited onboard memory (typical for quadruped robots) necessitates a small model footprint; our ELCIN reduces detection head parameters by 53%, contributing to a total model size of only 4.57 MB. Third, quadruped robots operate under varying viewpoints (e.g., crouching, climbing) and dynamic environments (changing lighting, occlusions), which make small object detection particularly challenging. To address this, we propose the Multi-Scale Edge-Spatial Feature Extraction Module (MSESTE) to enhance edge-aware features that are invariant to viewpoint changes, and the Adaptive Multi-Scale Contextual Feature Fusion Network (AMCFN) to dynamically integrate multi-scale information under varying conditions. Although object detection techniques based on deep learning have made remarkable progress in the field of intelligent inspection^8,9^, they still have high false detection and missed detection rates in complex environments, especially in small object detection tasks with multi-scale and dynamic background^10–12^.

First of all, the existing intelligent inspection system is difficult to cope with complex environmental changes, such as changes in lighting conditions, weather disturbances (such as rain, snow, fog, etc.) and the impact of background clutter^13,14^^.^ In these cases, traditional detection algorithms often fail to detect the target accurately and exhibit low robustness, especially when the target’s appearance is similar to the background. Secondly, in terms of small target detection, because the targets in the inspection environment may have very small physical sizes, the existing models often lack sufficient feature expression ability when capturing these small targets, resulting in serious problems of false detection and missing detection^15–17^. In addition, the intelligent inspection task usually requires the system to have high real-time and timely detection of potential risks, but the high computational complexity of the existing model makes it difficult to implement it on resource-constrained embedded devices^18–20^. These deficiencies highlight the small target detection problem of intelligent inspection in complex environments, which is still a key challenge to be solved. Therefore, we propose a series of improvement measures on the basis of YOLOv8n algorithm to further improve the detection accuracy, real-time performance and environmental adaptability.

To address the challenges of small object detection, various improved methods have been proposed in existing studies. For instance, Hu et al. introduced multi-scale feature fusion techniques in object detection to improve the detection accuracy of small objects^17^. In recent years, a series of studies on lightweight and small object detection have emerged, such as the SOD-YOLO framework based on YOLOv7-tiny^21^, the LAYN network incorporating multi-scale attention^22^, and AeroDetectNet designed for small objects in aerial remote sensing^23^, among others. Furthermore, comprehensive benchmark evaluations of the YOLO series models^24^ also provide important references for model selection and performance comparison. More recently, Shen et al. proposed a lightweight semantic feature extraction model with direction awareness for aerial traffic object detection^25^, demonstrating the growing interest in efficient feature representation for complex scenes. Additionally, beyond object detection, Li et al. introduced KACNet, which enhances CNN feature representation using Kolmogorov-Arnold networks for medical image segmentation^26^, exemplifying broader research efforts in lightweight CNN architectures. These advances further highlight the need for lightweight yet accurate models like MAE-YOLO for industrial inspection tasks. However, there remains a significant gap in practical strategies that simultaneously achieve extreme lightweight design and maintain high precision for small objects in complex industrial inspections. Existing approaches often struggle to optimize the trade-off between computational efficiency and feature representation power, particularly for multi-scale targets under dynamic backgrounds^27,28^. To address this issue, the multi-scale edge space feature extraction module (MSESTE) is proposed in our study, which optimizes the extraction of edge details and spatial features, allowing the model to more effectively detect small targets in complex backgrounds, thereby significantly enhancing detection accuracy in dynamic and complex environments.

The intelligent inspection systems also face the challenge of insufficient robustness in complex visual environments. Existing studies have attempted to enhance model robustness through the fusion of contextual information^29–31^, However, there remains significant room for improvement in the effective integration of features across different scales. To solve this problem, we propose an Adaptive Multi-Scale Contextual Feature Fusion Network (AMCFN), which achieves context fusion of features at different scales, significantly enhancing the model’s robustness in complex visual environments and ensuring stable and accurate detection in dynamic scenes.

Moreover, in the multi-scale target detection, the resource-constrained environment requires the detection algorithm to ensure the detection speed and maintain sufficient accuracy, which remains a significant challenge for the current deep learning methods^32–40^. Although lightweight improved models such as ECF-YOLOv7-Tiny^41^ and SCM-YOLO^42^ have emerged in recent years, along with methods like GAME-YOLO^43^ for UAV detection in low-visibility environments and PC-YOLO11s^44^ based on YOLO11, achieving high-precision, highly robust small object detection under constrained computational resources remains a critical and unresolved challenge in embedded deployment scenarios for industrial inspection. To address this issue, we propose an Efficient Layer-Shared Convolution with Independent Normalization (ELCIN), which adaptively coordinates the classification and regression tasks to ensure the high precision of multi-scale target detection while achieving a lightweight detection head. This approach bridges the gap in real-time performance and precision compared to existing methods.

In summary, we propose an intelligent inspection model named MAE-YOLO, specifically optimized for quadruped inspection robots to better meet the specific needs of intelligent inspection tasks. The model enhances detection accuracy and efficiency in inspection tasks while reducing computational overhead in resource-constrained environments. It provides more effective technical support for small object detection in complex scenarios such as power inspections and equipment fault detection. This paper details the development and implementation of these improved modules. To comprehensively evaluate the model’s performance, we employ a two-tiered validation strategy. First, we utilize the challenging VisDrone2019 dataset as a benchmark. While captured from a drone perspective, this dataset presents universal challenges critical to industrial inspection, such as small object detection, complex backgrounds, and varying lighting conditions, making it an ideal proxy for assessing fundamental robustness. Second, to more directly simulate industrial scenarios, we evaluate the model on a self-collected gesture dataset that encapsulates inspection-relevant challenges like occlusion and multi-target interaction under diverse backgrounds. This combined approach ensures that MAE-YOLO is rigorously tested for both general robustness and task-specific applicability. Experimental results demonstrate that these improvements achieve model lightweighting and high-accuracy detection, confirming the model’s practical value in real-time industrial inspection scenarios.

## Methods

**Fig. 1:**
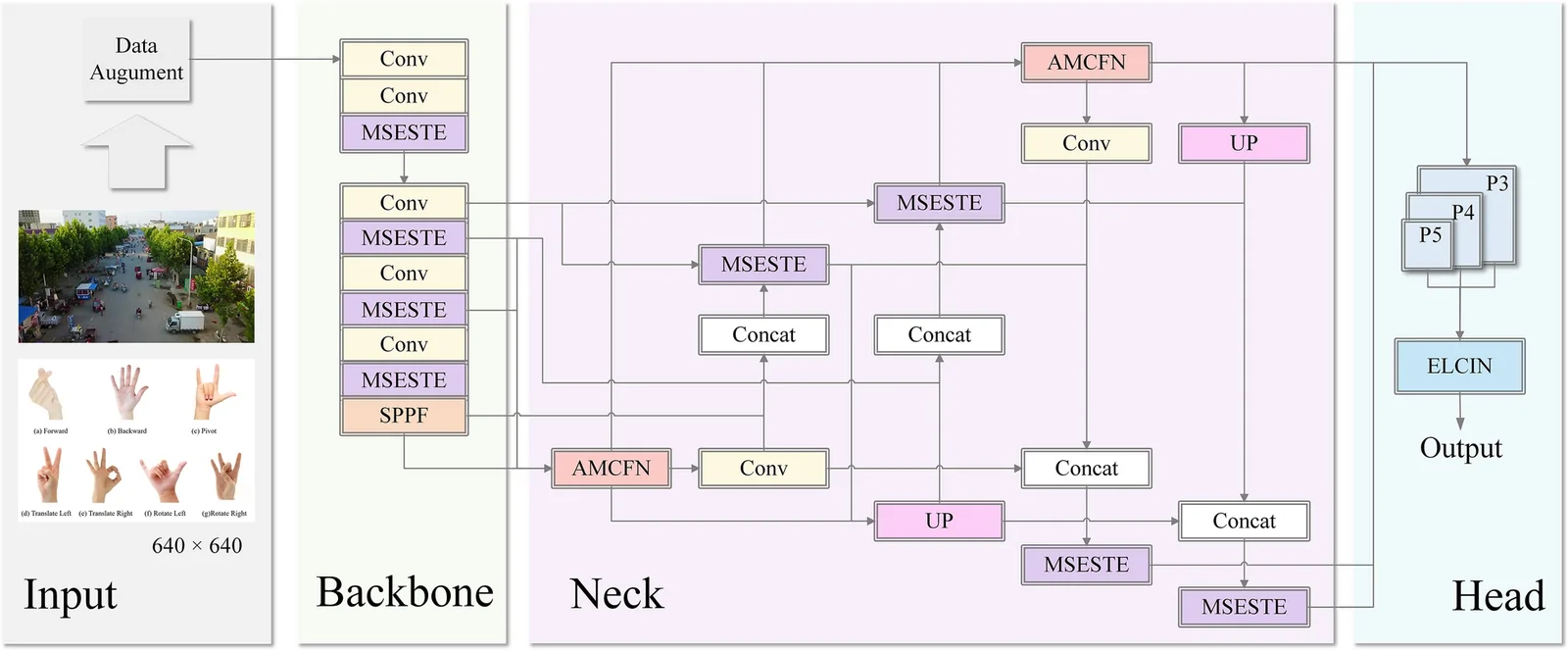
Overall structure of the MAE-YOLO.

The structural details of the improved YOLOv8n model, which we have named MAE-YOLO, is illustrated in Fig. 1. It primarily consists of three components: the backbone, the neck, and the head network. To provide a clear overview of our architectural improvements over YOLOv8n, Table 1 summarizes the key differences between each proposed module and its original counterpart. In the Backbone, we replace the original C2f. module in YOLOv8n with a MSESTE, which enhance feature extraction by capturing detailed and complex structural information at object edges. At the initial part of the Neck, after the first upsampling operation, we insert the AMCFN, which can fuse feature information from 8x, 16x, and 32× pooled output layers to fully integrate multi-scale context and enhance the detection capability of the model for objects of different sizes. Finally, we replace the original YOLOv8n detection head with our proposed ELCIN. This shared detection head improves computational efficiency through shared convolution operations while leveraging independent normalization to optimize the learning process of feature layers, thereby enhancing detection accuracy and efficiency.

**Table 1:** Comparison of proposed modules with original YOLOv8n components.

Component	Original YOLOv8n	MAE-YOLO (Ours)	Innovation
Backbone	C2f. module	MSESTE	Inject edge priors to enhance small-object boundary perception
Neck	Standard FPN	AMCFN	Dense multi-scale interaction with adaptive weights
Head	Independent Conv × 3 scales	ELCIN	Independent BN preserves scale specificity

### Multi-scale edge-spatial feature extraction module (MSESTE)

**Fig. 2:**
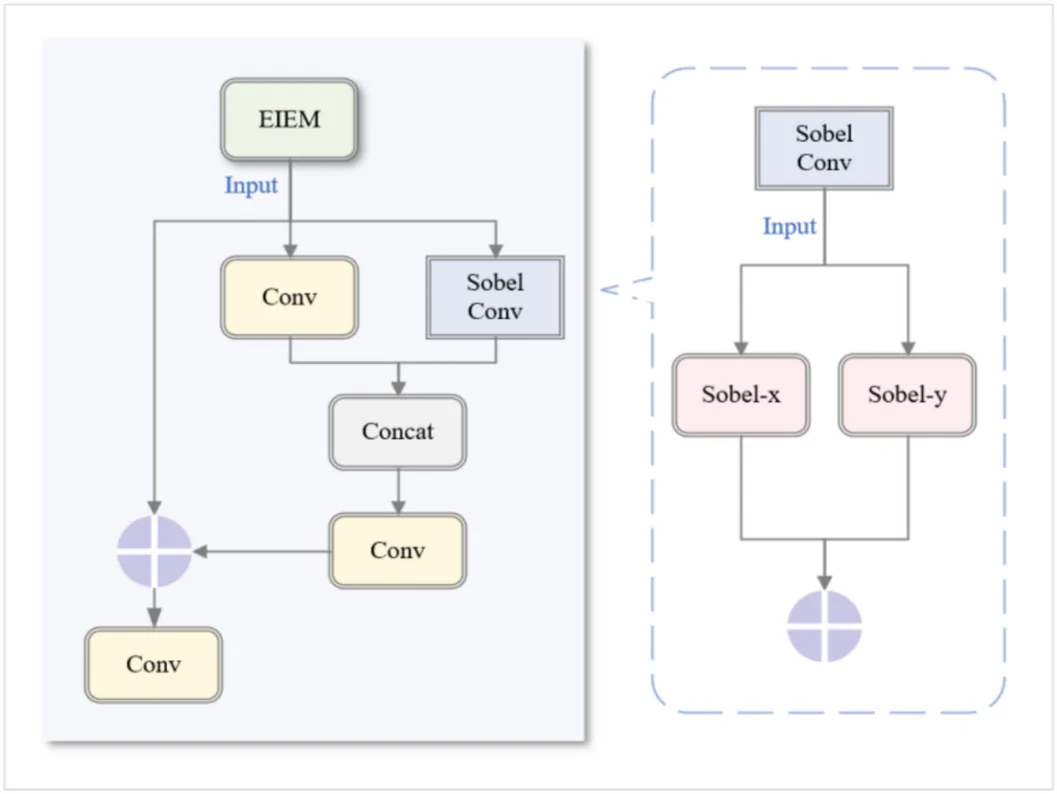
MSESTE structure.

We proposed an efficient module for extracting spatial and edge information, named the MSESTE, as shown in Fig. 2, given an input feature X, it is first divided into two parts: ${\mathrm{X}}_{{{\mathrm{skip}}}}$ and ${\mathrm{X}}_{{{\mathrm{eiem}}}}$. For ${\mathrm{X}}_{{{\mathrm{eiem}}}}$, it is processed by the EIEM module.${\mathrm{X}}_{{{\mathrm{skip}}}}$ is directly passed to the module’s output to preserve the original information. In the Edge Information Extraction Module (EIEM) module, the input feature ${\mathrm{X}}_{{{\mathrm{eiem}}}}$ is processed through a Sobel convolution branch and a convolutional feature branch, as described by Eqs. (1) and (2).

1 $${\mathrm{F}}_{sobel} = SobelConv(X_{eiem} )$$

2 $${\mathrm{F}}_{conv} = Conv(X_{eiem} )$$

The outputs of these two branches are concatenated, as shown in Eqs. (3).

3 $${\mathrm{F}}_{concat} = \left[ {F_{sobel} ,F_{conv} } \right]$$

Then, a 1 × 1 convolution layer is used for channel compression to obtain the fused feature, as described by Eqs. (4).

4 $$F_{compressed} = W_{c} F_{concat}$$

Next, the output of the EIEM module is merged with the original input ${\mathrm{X}}_{{{\mathrm{eiem}}}}$ through residual connection to form the final output feature, as shown in Eqs. (5).

5 $${\mathrm{F}}_{eiem} = F_{compressed} + X_{eiem}$$

Finally, the C2f_EIEM module concatenates $F_{{{\mathrm{eiem}}}}$ and ${\mathrm{X}}_{{{\mathrm{skip}}}}$ to obtain the final output feature, as described by Eqs. (6).

6 $$F_{output} = Concat(X_{skip} ,F_{eiem} )$$

This structural design enables the network to extract richer feature representations by effectively enhancing and fusing features while preserving the original information. The rationale behind combining Sobel operator with standard convolution is rooted in the specific challenges of small object detection in industrial inspection. Critical targets, such as structural cracks, surface scratches, or loose bolts, are often characterized by their high-frequency edge features. However, in cluttered industrial environments, these salient cues can be easily obscured by noise, low contrast, or varying illumination. The Sobel branch provides a strong, predefined inductive bias towards edge information, acting as a robust high-frequency filter that is less susceptible to being ‘washed out’ during initial training compared to learning edge detectors from scratch. This ensures that crucial structural boundaries are preserved and amplified from the outset. Concurrently, the standard convolutional branch learns complementary, data-driven texture and contextual features. The fusion of these pathways forces the network to explicitly prioritize and integrate edge information with semantic content, guiding the model to focus on the precise spatial locations and contours that define small objects. This is particularly vital when the target’s pixel area is limited, as its shape and outline become its most discriminative attributes, effectively countering the tendency of CNNs to lose fine-grained spatial details in deeper layers.

Comparative advantages of Sobel-based edge enhancement: Unlike learnable edge detectors (e.g., the first few convolutional layers in standard CNNs), the Sobel operator offers three distinct benefits for small object detection in industrial inspection. First, it is computationally efficient – Sobel convolution uses fixed, separable kernels (horizontal and vertical), requiring only two lightweight operations without backpropagation updates. Second, it provides illumination-invariant edge responses – because Sobel computes gradient magnitude, it is less affected by global brightness changes compared to data-driven features that may overfit to lighting conditions. Third, it offers explicit orientation sensitivity – the horizontal and vertical gradient maps can be used separately or combined, which is particularly useful for detecting elongated small objects (e.g., cracks, wires). In contrast, learnable edge detectors require significant training data to approximate similar behavior and often fail to generalize under domain shifts. While alternative handcrafted detectors such as Canny offer richer edge information (non-maximum suppression, hysteresis thresholding), they are computationally more expensive and produce binary edge maps that discard gradient magnitude information. Sobel strikes an optimal balance between efficiency and information richness, making it preferable for real-time embedded deployment. To the best of our knowledge, our work is the first to systematically integrate Sobel with modern CNN backbones for small object detection in industrial inspection, and we provide ablation studies (see Section "Ablation analysis based on VisDrone2019 dataset", models with/without MSESTE) that confirm its contribution. To further justify the choice of Sobel, we compare it with alternative edge detection methods in terms of computational cost and edge quality for small object detection in embedded scenarios. Canny produces cleaner, thin edges but requires additional steps (non-maximum suppression, hysteresis thresholding), which incur higher latency and are less parallelizable on GPUs. Laplacian is sensitive to noise and tends to produce double edges, potentially confusing the localization of small objects. Learnable edge modules (e.g., edge-aware convolutions, trainable Gabor filters) offer adaptability but introduce extra parameters and may overfit to domain-specific edge patterns, reducing generalization across different industrial environments. In contrast, the Sobel operator strikes an optimal balance: it uses fixed, separable kernels (computationally efficient, no backpropagation), provides gradient magnitude and orientation information, and is robust to illumination changes. This makes Sobel particularly suitable for real-time, resource-constrained deployment on quadruped robots.

### Adaptive multi-scale contextual feature fusion network (AMCFN)

**Fig. 3:**
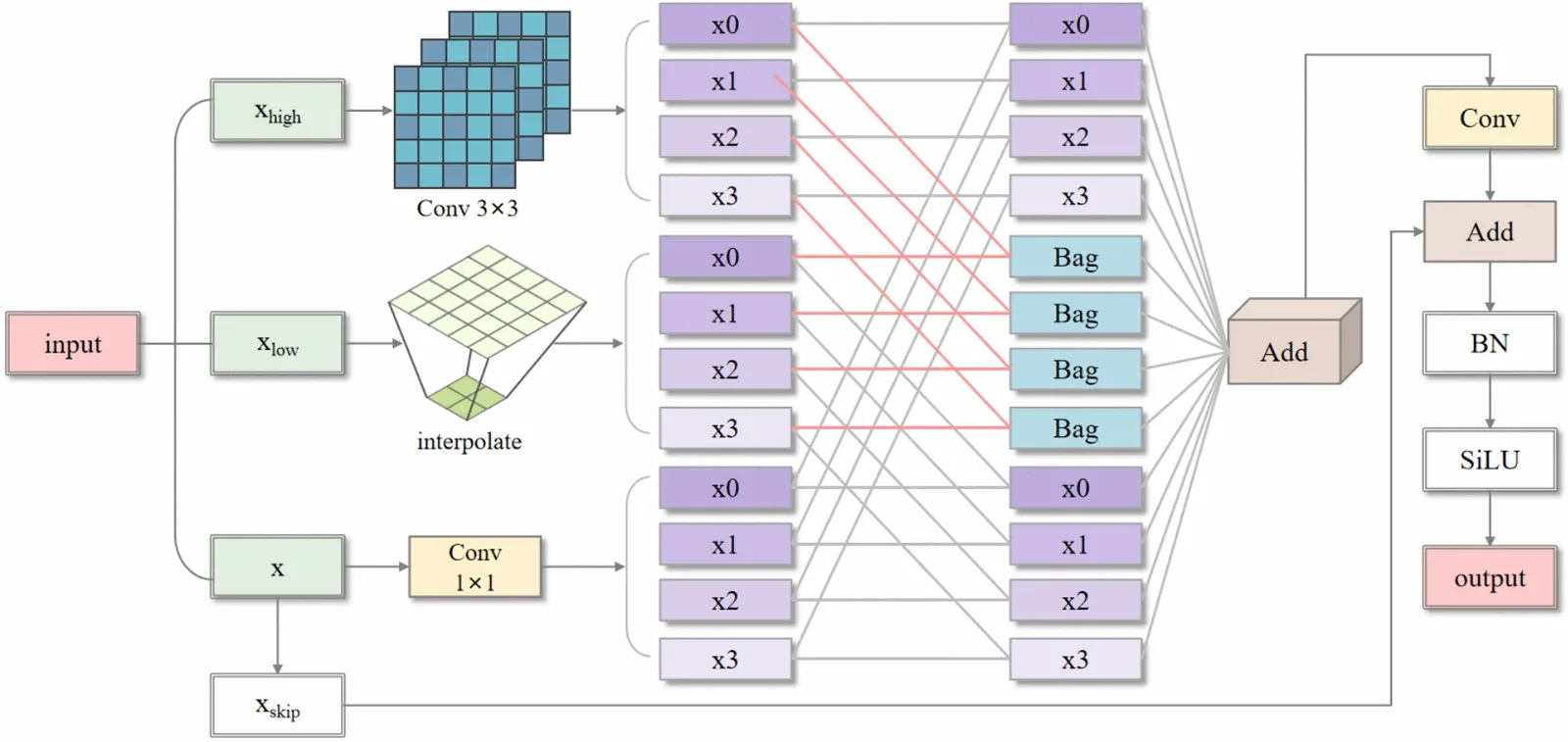
AMCFN structure.

To enhance the multi-scale contextual information for feature extraction, we introduced a new architecture called the AMCFN in the neck network. The design of this module includes a Bag-weighted fusion mechanism, feature skip connections, and output processing through a tail convolutional layer, as shown in Fig. 3. The inputs are the 8x, 16x, and 32× downsampled output layers, which correspond to high-resolution feature map *x*_*high*_, medium-resolution feature map x, and low-resolution feature map *x*_*low*_, respectively. The low-resolution feature map is passed through a *1* × *1* convolutional layer to reduce the number of channels, followed by an upsampling operation to match the resolution of the intermediate feature map x, as described in Eq. (7).

7 $${x}_{low}= \mathrm{I}\mathrm{n}\mathrm{t}\mathrm{e}\mathrm{r}\mathrm{p}\mathrm{o}\mathrm{l}\mathrm{a}\mathrm{t}\mathrm{e} \left({Conv}_{1\times 1}({x}_{low})\right)$$

The high-resolution features are passed through a convolutional layer with a kernel size of *3* × *3*, and a stride of 2 and dilation of 2 are applied to expand the spatial features. After convolution, the high-resolution features are further divided into four groups for progressive fusion with other resolution features. The intermediate features undergo a *1* × *1* convolution to generate the base features for the skip connection. Meanwhile, these features are also split into four groups to be combined with subsequent features from different scales, as described in Eq. (8):

8 $${x =Conv}_{1\times 1}\left(x\right),{x}_{skip}=x$$

The core of the AMCFN is the Bag Selective Fusion Unit, which enables the adaptive fusion of features from different scales using an attention mechanism, achieving efficient information propagation. The fusion strategy can be represented by Eq. (9):

9 $$y = \sigma \left( d \right) \cdot p + \left( {1 - \sigma \left( d \right)} \right) \cdot i$$

where, *p* represents high-resolution or low-resolution features, *i* represents the intermediate-resolution features that are fused with *p*, *d* represents the attention signal obtained after extracting intermediate features, *σ(·)* is the Sigmoid function, used to generate the fusion weights. Specifically, the attention signal *d* is generated by applying a lightweight convolutional layer (Conv_att with a *3* × *3* kernel) to the concatenated intermediate features from the three scales (*x*_*high*_, *x*, *x*_*low*_), followed by a Sigmoid activation to produce pixel-wise spatial attention weights. In formula: d = *σ*(Conv_att([*x*_*high*_, *x*, *x*_*low*_])). This produces spatially-varying weights that adaptively blend features at each pixel location, enabling the network to emphasize high-resolution details near object boundaries and low-resolution context in homogeneous regions. Unlike channel-wise attention, our spatial attention mechanism is more suitable for scale-selection tasks where the optimal scale varies across spatial positions. This Bag-weighted fusion mechanism constitutes a key departure from traditional feature pyramid networks (FPNs). While standard FPNs rely on a fixed, unidirectional (top-down) fusion path with operations like element-wise addition, our approach introduces a dynamic, content-aware weighting scheme. The core innovation lies in generating pixel-wise attention weights *σ*(*d*) directly from the intermediate features themselves (Eq. 9). This allows the network to adaptively determine, for each spatial location, the relative importance of features from different scales. For instance, at the boundary of a small object, the mechanism can learn to assign higher weights to the high-resolution feature map to preserve detail, while in a homogeneous region requiring contextual understanding, it can shift focus to the low-resolution, semantically rich features. This stands in contrast to methods like ASFF, which often use fixed or learned but non-adaptive weights. Our Bag unit enables a more flexible and context-dependent feature fusion, which is crucial for maintaining detection stability in the dynamic and multi-scale environments encountered during mobile inspection. After being processed by the Bag Unit, the high, medium, and low-resolution features generate four sub-features, denoted as ${\mathrm{x}}_{0} ,{\mathrm{x}}_{1} ,{\mathrm{x}}_{2} ,{\mathrm{x}}_{3}$. These features are then concatenated along the channel dimension (Eq. 10), and passed through a 1 × 1 convolutional layer (Eq. 11) at the tail, for channel integration. The fused features are then added to the original intermediate feature x_skip through a skip connection (Eq. 12), to enhance gradient flow and feature reuse. Finally, the fused features undergo Batch Normalization (BN) and a SiLU activation function, as shown in Eq. 13, to generate the final output.

10 $${x}_{fused} =\mathrm{C}\mathrm{o}\mathrm{n}\mathrm{c}\mathrm{a}\mathrm{t} {(x}_{0},{x}_{1} ,{x}_{2} ,{x}_{3})$$

11 $${x}_{tail}={Conv}_{1\times 1}({x}_{fused})$$

12 $${x}_{out}={x}_{tail}{ + x}_{\mathrm{s}\mathrm{k}\mathrm{i}\mathrm{p}}$$

13 $${x}_{output}=\mathrm{S}\mathrm{i}\mathrm{L}\mathrm{U}(\mathrm{B}\mathrm{N}{(x}_{out}))$$

### Efficient layer-shared convolution with independent normalization (ELCIN)

**Fig. 4:**
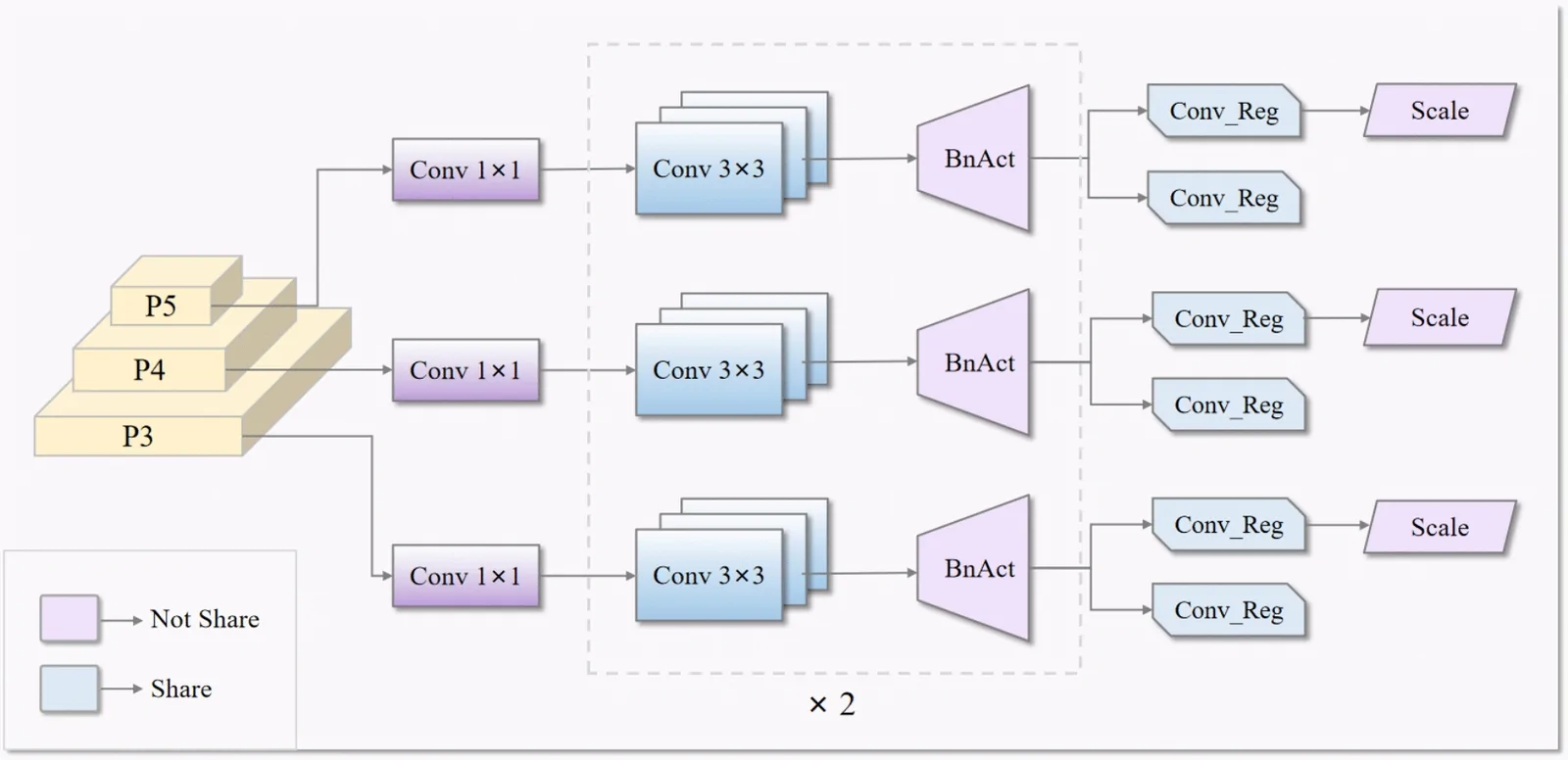
ELCIN structure.

The introduction of ELCIN can enhance both the accuracy and computational efficiency of object detection models. This module integrates concepts such as convolution sharing, independent feature normalization, and lightweight design to effectively balance computational complexity and detection performance. Specifically, the module adopts a strategy of shared convolution combined with independent Batch Normalization to reduce parameter redundancy during feature fusion, thereby improving the feature representation capabilities of the model, making it particularly suitable for resource-constrained environments. The key components are illustrated in Fig. 4. First, the input feature maps from different levels (P3, P4, P5) are compressed using a 1 × 1 convolution to ensure that each scale of feature maps has the same feature dimension, as described in Eq. (14):

14 $$X_{i}{\prime} = Conv_{1 \times 1} \left( {X_{i} } \right),\,\,\,\,\,i = 3,4,5$$

Secondly, two shared convolutions (each with a 3 × 3 kernel) are used to extract the input features. After each shared convolution, Batch Normalization and an activation function are applied independently to each input feature map, as shown in Eq. (15), where *σ* represents the activation function (SiLU is used in this paper).

15 $$X_{i}^{{(j + 1)}} = BN_{{i,j}} (\sigma (Conv_{{shared}} (X_{i}^{{(j)}} ))),\,j = 1,2$$

The features processed by the shared convolution are then passed through two separate convolutional layers for regression prediction (Conv_Reg) and classification prediction (Conv_Cls), as described in Eqs. (16) and (17). The regression branch is responsible for predicting the coordinates of the bounding boxes, outputting four coordinate values, while the classification branch predicts the class score for each candidate box. Each regression branch uses an additional scale adjustment layer to further refine the bounding box scale.

16 $$R_{i} = Scale_{i} (Conv_{{{\mathrm{Re}} g}} (X_{i}^{(2)} ))$$

17 $${\mathrm{C}}_{{\mathrm{i}}} = Conv_{cls} (X_{i}^{(2)} )$$

Finally, Distribution Focal Loss (DFL) is used to decode the bounding boxes, as described in Eq. (18), helping the model learn the finer positions of the targets during bounding box prediction.

18 $${\mathrm{Y}}_{i} = Concat(R_{i} ,C_{i} )$$

The primary driver for model lightweighting in our method is this architectural redesign within the ELCIN module. The key innovation lies in the strategy of sharing convolutional kernels across the feature maps from different pyramid levels (P3, P4, P5) for feature extraction. This significantly reduces the number of parameters required in the detection head, which is traditionally one of the most parameter-heavy components in object detectors. Crucially, to compensate for the potential loss of discriminative power due to kernel sharing, ELCIN employs independent Batch Normalization (BN) layers for each scale. This allows the model to learn scale-specific distribution statistics, preserving the unique characteristics of features at each level and ensuring that the shared convolutions do not lead to a significant performance drop.

Theoretical justification: Sharing kernels across scales is justified by the observation that the fundamental operations for object localization and classification (e.g., edge detection, corner detection) are scale-invariant; only the statistical distributions of features differ across scales. By sharing kernels, the network learns common transformation patterns, while independent BN layers handle scale-specific normalization. This design reduces detection head parameters by 53% (from 1.20M to 0.56M) compared to YOLOv8n, contributing to a 24% overall model size reduction. Compared to other lightweight heads (e.g., PicoDet’s shared head, PP-YOLOE’s ESE block), ELCIN is simpler, requires no extra attention modules, and achieves superior accuracy-efficiency trade-off.

## Experimental results

To validate the effectiveness of MAE-YOLO for intelligent inspection, we designed experiments to answer two key questions: (1) Can the model achieve high accuracy and robustness on complex, small-object detection tasks that share challenges with industrial environments? (2) Can the model maintain efficiency suitable for resource-constrained deployment? The following sections detail the datasets and experiments designed to answer these questions.

### Dataset

Publicly available dataset: VisDrone2019 Dataset. The VisDrone2019 dataset is one of the most widely used drone image datasets for object detection and tracking in complex outdoor environments. It contains over 10,000 high-resolution images and 96 video sequences captured by drones at various locations, encompassing a wide range of complex and challenging scenes. These data samples include more than 540,000 annotated objects, divided into 10 different categories such as pedestrians, cars, buses, and bicycles. The images and videos were taken under diverse conditions, including variations in altitude, shooting angles, and environmental complexity. Due to factors such as differences in object size, occlusions, lighting variations, cluttered backgrounds, and interference from visually similar objects, the VisDrone2019 dataset provides a valuable benchmark for research on small object detection in intelligent inspection.

To quantify the challenge of small object detection, we analyzed the object size distribution in the VisDrone2019 dataset. We define small objects as those with an area less than 32 × 32 pixels. Over 40% of the annotated objects in VisDrone2019 fall into this category, with many critical targets (e.g., distant pedestrians, small vehicles) being as small as 10 × 10 pixels. The performance on these small objects is a key focus of our evaluation.

Self-collected dataset. The self-collected multi-object gesture dataset is one of the most comprehensive datasets for evaluating detection performance in complex multi-object scenarios. It consists of approximately 1,400 images, each containing 5 to 7 individuals displaying random gestures from a set of seven categories (Forward, back, turn in place, pan left, pan right, rotate left, rotate right, as shown in Fig. 1), ensuring diversity and randomness across samples. All images were manually annotated using bounding boxes for the seven gesture categories by three trained annotators; the final label for each instance was determined by majority vote, yielding a high inter-annotator agreement. These images were captured in diverse, inspection-relevant environments, including equipment rooms, educational areas, sports fields, outdoor open spaces, industrial facilities, and densely vegetated regions. To further enhance variability, the background changes every 100 images, with a mix of static and dynamic scenes under different lighting conditions, fully simulating the complex operational environments encountered in real-world industrial inspections. The dataset includes typical challenges, such as random occlusions between objects and gesture objects blending with complex backgrounds, exhibiting small-object characteristics. The seven gesture categories represent typical human–robot interaction commands (e.g., forward, back, turn left, turn right, etc.) required for controlling quadruped robots during industrial inspection tasks, such as navigating through narrow passages or signaling equipment status. Three trained annotators used the LabelImg software to annotate the bounding boxes. The final label for each instance was determined by majority vote. The dataset comprises annotated instances across seven categories, each containing more than one thousand instances, ensuring a balanced distribution. This data set can be used to evaluate the performance of the model in chaotic, inspection-like complex environments, and provides a valuable benchmark for testing the adaptability, robustness and detection accuracy of the model in real complex scenarios. In the experiment, the rest images are divided into training and validation sets in a ratio of 8: 2.

### Environment setup

The experimental environment is based on the Ubuntu 20.04 LTS operating system, utilizing a 24 GB NVIDIA GeForce RTX 3090 GPU for hardware acceleration. The model is run in a Python 3.10 environment. The key parameter settings include 600 epochs, a batch size of 32, an initial learning rate (lr0) of 0.01, a learning rate factor (lrf) of 0.01, momentum set to 0.937, and a weight decay of 0.0005.

### Experiment and analysis

In this paper, ablation experiments and comparison experiments are executed.

#### Ablation analysis based on VisDrone2019 dataset

Table 2 describes the results of ablation experiments for different models, including validation datasets and test datasets. From the validation results, it can be seen that the MAE-YOLO shows significant advantages across multiple performance metrics. Firstly, the comprehensive mAP@50 value of MAE-YOLO reaches 35.2%, significantly higher than that of other models, demonstrating its superiority in overall detection accuracy. While the absolute improvement of 2.6% in mAP@50 over YOLOv8n may appear modest, this enhancement is achieved on the notoriously challenging VisDrone2019 dataset, where state-of-the-art models often plateau with marginal improvements due to extreme variations in object scale, viewpoint, and background complexity. Additionally, MAE-YOLO also shows balanced detection performance across different object sizes (small, medium, and large). Specifically, it achieves 10.9%, 30.5%, and 36.2% for small (S), medium (M), and large (L) objects, respectively. This balance indicates that MAE-YOLO is well-suited for complex, multi-object scenarios, with strong detection capabilities across a wide range of object sizes.

**Table 2:** Ablation experiment results with different modules.

Baseline	Method			Precision (%)	Recall (%)	mAP50 (%)	Size(MB)	parameters	gradients	S (%)	M (%)	L (%)
	MSESTE	AMCFN	ELCIN									
YOLOv8n^Val^	√			45.8	33.2	34.3	6	2,858,238	2,858,222	9.8	28.6	40.1
		√		47.5	33.9	34.7	5.47	2,743,694	2,743,678	9.9	29.4	39.3
			√	44.9	33.3	33.3	5	2,368,045	2,368,029	9.5	28.6	39.5
	√	√		46.2	34.3	35	5.4	2,589,134	2,589,118	10.1	29.3	39.7
	√		√	44.7	31.8	32.6	4.7	2,213,485	2,213,469	9.8	28.6	40.1
		√	√	44.4	31.6	32.2	4.8	2,307,837	2,307,821	8.9	27.9	36.8
MAE-YOLO^Val^	√	√	√	46	35	**35.1**	4.57	2,260,157	2,260,141	10.9	30.5	36.2
YOLOv8n^Test^	**√**			38.6	29.1	26.8	6	2,858,238	2,858,222	5.7	22.1	32.2
		**√**		40.8	29.8	27.7	5.47	2,743,694	2,743,678	6	23.6	34.8
			**√**	39.8	28.4	26.2	5	2,368,045	2,368,029	5.6	21.9	34.1
	**√**	**√**		40.7	27.9	26	4.7	2,213,485	2,213,469	5.3	21.6	32.3
	**√**		**√**	39.7	29.8	27.6	5.4	2,589,134	2,589,118	5.9	23	35.2
		**√**	**√**	38.4	27.8	25.9	4.8	2,307,837	2,307,821	5.1	21.9	33.5
MAE-YOLO^**Test**^	**√**	**√**	**√**	41	30.1	27.6	4.57	2,260,157	2,260,141	6.4	23.6	32.4

Bold values indicates the best performance among the compared models.

In comparison, the MSESTE-YOLOv8n module performed exceptionally well for large object detection, achieving an mAP@50 of 40.1% for large targets, making it ideal for applications where high accuracy in large object detection is crucial. However, despite its improvements in large-object performance, it fell short in detecting small and medium-sized objects compared to MAE-YOLO. Similarly, the AMCFN-YOLOv8n module improved in terms of precision and medium-object detection, reaching a precision of 47.5%, but its overall mAP@50 remained slightly lower than MAE-YOLO, suggesting its adaptability in multi-category scenarios is relatively limited.

For models with combined modules, MSESTE-AMCFN-YOLOv8n approached MAE-YOLO’s performance, reaching an mAP@50 of 35%, but this came at the cost of increased model parameters and complexity, resulting in a bulkier model with higher computational requirements. Likewise, MSESTE-ELCIN-YOLOv8n modules showed good performance in large-object detection, but its overall recall was lower, and the mAP@50 did not match that of MAE-YOLO, indicating that multiple module combinations failed to achieve an optimal balance between performance and model complexity.

Similarly, the same conclusion can be drawn from the test set results (the last seven rows of Table 2), MAE-YOLO achieves an effective balance between detection performance, model size, and parameter count. It maintains high precision and recall rates while keeping computational complexity low, resulting in strong detection performance across small, medium, and large objects. Compared to other models, MAE-YOLO provides superior stability and adaptability for multi-category and multi-size object detection tasks without adding extra parameters or complexity, making it the best-performing improved model in this ablation study. This makes MAE-YOLO highly suitable for real-world applications in complex multi-object detection scenarios.

#### Comparative analysis based on VisDrone2019 dataset

**Fig. 5:**
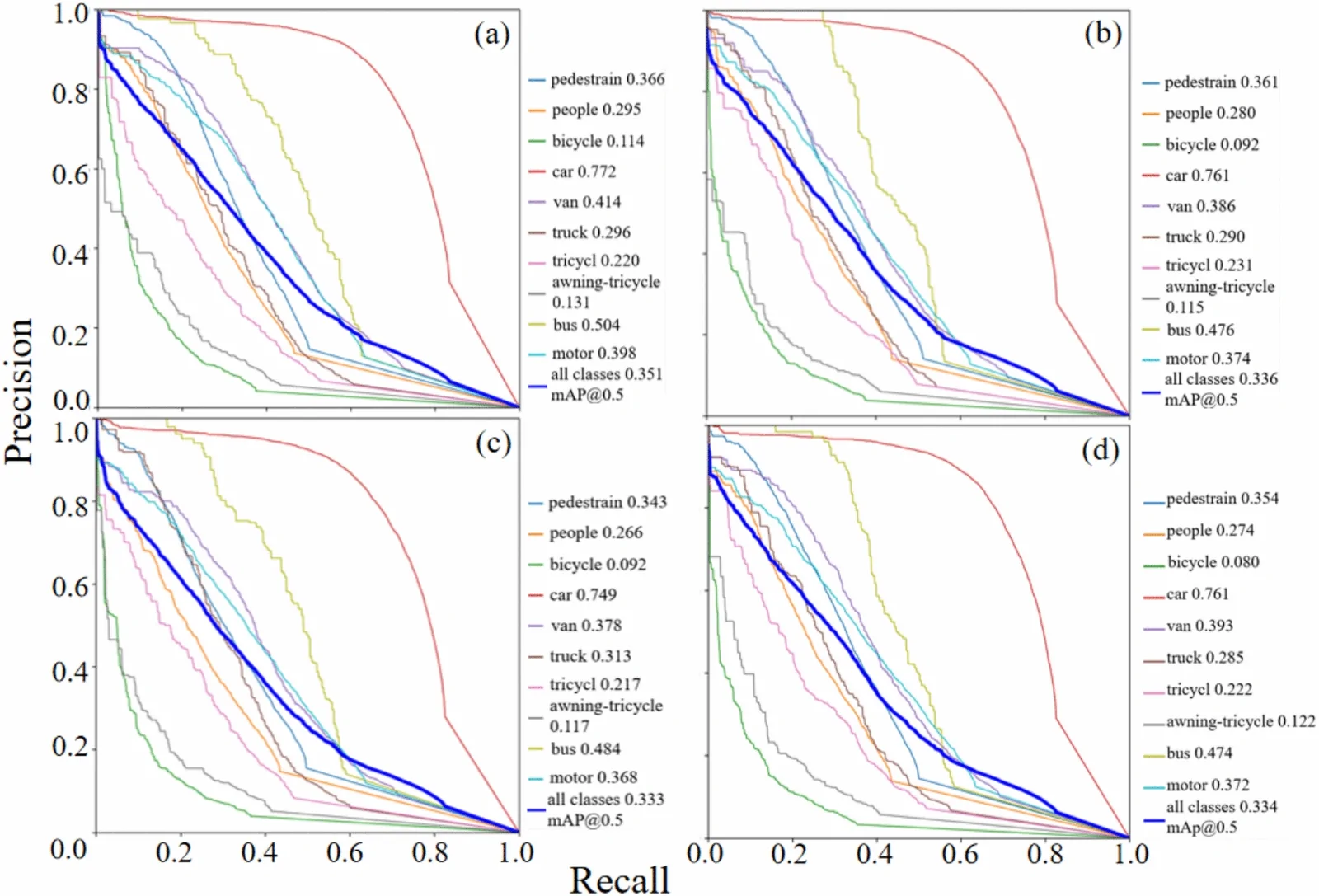
The precision-recall curve for different models. (a) MAE-YOLO; (b) YOLOv8n; (c) YOLOv10n; (d) YOLOv11n.

Based on the VisDrone2019 dataset, we compared the original YOLOv8n, YOLOv10n and YOLOv11n with the improved MAE-YOLO model, as shown in Fig. 5. It is evident that MAE-YOLO achieves the highest overall mAP@0.5 value, reaching 0.351, as shown in Fig. 5(a), significantly surpassing the YOLOv8n, YOLOv10n, and YOLOv11n models. This indicates that MAE-YOLO can achieve higher precision and recall in complex backgrounds and small-object detection. Although YOLOv8n performs well in tricycle, as shown in Fig. 5(b), its overall mAP@0.5 value is lower than that of MAE-YOLO. This suggests that YOLOv8n does not maintain stable precision and recall in small-object detection and complex background scenarios. While YOLOv10n’s curve appears relatively balanced, its mAP@0.5 value is only 0.333 in Fig. 5(c). Both precision and recall are low across multiple categories, particularly for small-object classes like pedestrians and people, where significant missed detections occur. Figure 5(d) shows that YOLOv11n achieves a slightly higher mAP@0.5 value of 0.334 compared to YOLOv10n. However, the distribution of category curves is uneven, and the model struggles to maintain a balance between precision and recall. Specifically, its precision rapidly drops under high recall conditions. In contrast, MAE-YOLO demonstrates smoother and more balanced curves, with minimal differences in detection capabilities across categories. This advantage makes it more suitable for multi-class detection tasks. In the following sections, we will further elaborate on the advantages of the MAE-YOLO model through comparisons across different evaluation metrics.

Based on the VisDrone2019 dataset, we performed a comparative analysis of the original YOLOv8n, YOLOv10n, YOLOv11n, YOLOv12n, and MAE-YOLO model parameters, as shown in Table 3. YOLOv12n is the latest version in the YOLO series, featuring an attention-based backbone and reparameterized detection head for improved accuracy-efficiency trade-offs. We include it as a baseline to compare MAE-YOLO against the most recent YOLO variant. The first five rows represent the results on the validation dataset, while the latter five rows present the results on the test dataset. From the validation dataset results, it is evident that MAE-YOLO exhibits significant advantages across multiple metrics, with a precision of 46%, recall of 35%, and mAP@50 of 35.1%, all of which outperform the other models. This indicates that MAE-YOLO achieves superior detection performance compared to other models. Moreover, MAE-YOLO has the smallest model size, parameter count, and gradient count, with values of 4.57 MB, 2,260,157, and 2,260,141, respectively, showcasing its higher computational efficiency and lightweight characteristics. In terms of detection performance across different object sizes, MAE-YOLO outperforms other models for small(S) and medium(M) sized objects, achieving 10.9% and 30.5%, respectively. This demonstrates its superior adaptability in small-object detection tasks within complex scenarios. Similarly, from the test results, although all models showed a decrease in parameter metrics, MAE-YOLO still maintains superior performance and efficiency, making it the best choice for multi-object detection tasks.

**Table 3:** Comparative analysis results with different modules.

Baseline	Precision (%)	Recall (%)	mAP50 (%)	Size(MB)	Parameters	Gradients	S (%)	M (%)	L (%)
YOLOv8n^**Val**^	43.4	33.6	33.6	5.97	3,012,798	3,012,782	9.6	28.1	37.1
YOLOv10n^**Val**^	44.6	34.3	33.3	5.5	2,710,940	2,710,924	10	28.1	34.4
YOLOv11n^**Val**^	43.1	33.4	33.4	5.23	2,591,790	2,591,774	9.6	27.6	34.9
YOLOv12n^**Val**^	43.5	33.1	32.5	5.5	2,558,678	2,558,093	9.2	27	35.6
MAE-YOLO^**Val**^	**46**	**35**	**35.1**	**4.57**	**2,260,157**	**2,260,141**	**10.9**	**30.5**	**36.2**
YOLOv8n^**Test**^	38.9	29.7	27.1	5.97	3,012,798	3,012,782	5.6	22.9	33.3
YOLOv10n^**Test**^	39	30.1	27.6	5.5	2,710,940	2,710,924	6.3	21.9	28.6
YOLOv11n^**Test**^	38.4	29.5	26.9	5.23	2,591,790	2,591,774	5.9	22.1	36.1
YOLOv12n^**Test**^	39.2	29.5	27.1	5.5	2,558,678	2,558,093	5.6	22.2	34.8
MAE-YOLO^**Test**^	**41**	**30.1**	**27.6**	**4.57**	**2,260,157**	**2,260,141**	**6.4**	**23.6**	**32.4**

Bold values indicates the best performance among the compared models.

#### Analysis based on self-collected dataset

In intelligent inspection tasks, small-object gesture recognition is often required for human–machine interaction. Therefore, we also collected a multi-target gesture dataset to evaluate the model’s performance in complex scenarios. As shown in Table 4, it provides a comparative analysis and ablation study of different algorithm models on the self-collected gesture dataset. The first seven rows represent the experimental results with different improvement methods (i.e., ablation analysis), while the last four rows show the comparison between different models. From the ablation experiments (the first seven rows), it can be observed that although adding a single improvement module or combining multiple modules results in similar precision, recall, and mAP@50 values. However, the model size, parameter and gradients size of these models are much larger than those of the MAE-YOLO. This highlights the efficiency of ours model in terms of model size and complexity.

**Table 4:** Ablation and Comparative analysis results with different modules based on Self-collected dataset.

Baseline	Method			Precision (%)	Recall(%)	mAP50 (%)	Size(MB)	parameters	gradients	S (%)	M (%)	L (%)
	MSESTE	ELCIN	AMCFN									
YOLOv8n	**√**			95.9	95.3	97.4	6	2,857,653	2,857,637	/	55	59.9
		**√**		95.2	95.9	97.4	5	2,857,653	2,857,637	/	65.2	58.5
			**√**	95.3	96.4	97.3	5.7	2,743,109	2,743,093	/	60.2	59.7
	**√**		**√**	93.9	95.6	97.4	5.4	2,265,450	2,265,434	/	55.1	58.7
	**√**	**√**		95.8	95.3	97.7	5.3	2,530,949	2,530,933	/	60.1	59.9
		**√**	**√**	92.6	95.9	96.9	4.8	2,307,642	2,307,626	/	60.1	58.1
MAE-YOLO	**√**	**√**	**√**	94.9	96.3	97.6	**4.7**	2,184,570	2,184,554	/	52.5	59.2
YOLOv8n	/	/	/	95.3	95.5	97.4	6.2	3,012,213	3,012,197	/	50.2	59.6
YOLOv10n	/	/	/	95.2	94.9	97.6	5.5	2,710,940	2,710,924	/	50.2	59.6
YOLOv11n	/	/	/	95.2	95.1	97.1	5.24	2,591,010	2,590,994	/	45.1	60.1
YOLOv12n	/	/	/	95.9	94.6	97.2	5.5	2,558,093	2,558,093	/	62.5	60.2

Bold values indicates the best performance among the compared models.

According to the comparison experiment (the last four rows), although YOLOv8n, YOLOv10n, and YOLOv11n also achieve high precision, recall, and mAP@50 values. However, in terms of computational efficiency, MAE-YOLO stands out with a model size as small as 4.7 MB, which is reduced by 24% (from 6.2 MB to 4.7 MB) compared with YOLOv8n. This dual achievement of enhanced accuracy coupled with increased lightweightness is a non-trivial outcome in model design, addressing the critical need for efficiency in embedded deployment. Its parameters and gradient size are 2,184,570 and 2,184,554 respectively, which are obviously smaller than other models. Therefore, through the self-collected data set, we can still see that MAE-YOLO has achieved a better balance in detection performance, model size and parameter number, which not only ensures high precision and high recall rate, but also maintains low computational complexity, making it perform better in different types of target detection.

#### Comparison with a broader range of models

To further validate the competitiveness of MAE-YOLO beyond the YOLO and address the scope of industrial inspection, we extended our comparisons to include a wider range of state-of-the-art object detectors. We selected representative models from different paradigms: FCOS and CenterNet, as well as the latest baseline models from the YOLO series for comparison.The comparative results on the VisDrone2019 dataset are summarized in Table 4.

From Table 5, it can be observed that MAE-YOLO achieves the highest mAP@50 on the VisDrone2019 dataset among all compared methods. More importantly, it accomplishes this while maintaining the smallest model size. Although FCOS and CenterNet are strong general-purpose detectors, their larger model sizes make them less suitable for resource-constrained inspection devices. These results comprehensively demonstrate that MAE-YOLO strikes an superior balance between detection accuracy, model efficiency, and generalizability, making it highly suitable for a variety of intelligent inspection applications.

**Table 5:** Comparison with broader state-of-the-art methods.

	FCOS	CenterNet	YOLOv8n (baseline)	YOLOv13n (baseline)	MAE-YOLO (Ours)
mAP50(%)	31.5	29.8	33.6	32.0	35.1
Size(MB)	12.1	14.5	5.97	5.2	4.57

#### Comparative analysis with the latest lightweight method SOD-YOLO

To further validate the competitiveness of MAE-YOLO in lightweight small object detection tasks, we compared it with the recently proposed lightweight method SOD-YOLO^21^ on the VisDrone2019 dataset. SOD-YOLO, also focused on addressing small object detection, is one of the representative lightweight frameworks in this field.

Table 6 details the comparative results of the two methods in terms of AP@0.5 for each category and the overall mAP@0.5 on the VisDrone2019 validation set. It can be clearly observed that MAE-YOLO outperforms SOD-YOLO on 9 out of the 10 categories. Particularly for key and challenging categories such as “pedestrian”, “people”, “car”, “van”, “bus”, and “motor”, MAE-YOLO achieves significant precision improvements (with an average increase of over 7 percentage points). For instance, on the “car” category, MAE-YOLO’s AP@0.5 reaches 77.2%, representing a substantial absolute improvement of 16.0% compared to SOD-YOLO’s 61.2%; similarly, a significant increase from 28.9% to 39.8% was achieved on the “motor” category.

**Table 6:** Comparison of MAE-YOLO and SOD-YOLO on VisDrone2019 Set (AP@0.5(%)).

Models	pedestrian	people	bicycle	car	van	truck	tricycle	awning-tricycle	bus	motor	mAP@0.5
SOD-YOLO^21^	29.1	19.9	11.3	61.2	37.7	31.2	21.1	11.8	48.6	28.9	30.08
MAE-YOLO (ours)	36.6	29.5	11.4	77.2	41.4	29.6	22.0	13.1	50.4	39.8	35.1

More importantly, the overall detection accuracy of MAE-YOLO (mAP@0.5 of 35.1%) significantly surpasses that of SOD-YOLO (mAP@0.5 of 30.08%), with an absolute improvement of about 5 percentage points. This result strongly demonstrates that, although both are lightweight designs, MAE-YOLO, through its innovative MSESTE, AMCFN and ELCIN. More effectively addresses the challenges of feature extraction, representation, and fusion for small objects in complex backgrounds while maintaining a lightweight model size (4.57 MB), thereby achieving superior detection performance.

## Conclusion

In summary of the above experimental analyses, MAE-YOLO demonstrates outstanding performance across multiple evaluation settings. Compared to baseline models (such as YOLOv8n-13n), it achieves dual improvements in both accuracy (mAP@0.5) and model lightweighting (parameter count and size). When compared to broader detection frameworks (e.g., FCOS, CenterNet), it achieves a superior balance between accuracy and efficiency. In particular, when compared with the state-of-the-art contemporary lightweight method specifically designed for small object detection, SOD-YOLO, MAE-YOLO exhibits clear advantages in the vast majority of detection categories as well as in overall accuracy. These results fully validate the effectiveness and advancement of MAE-YOLO’s overall architectural design, demonstrating that its proposed modules–-MSESTE, AMCFN, and ELCIN–-work in synergy to better address the core challenges of small object detection in practical scenarios such as industrial inspection.

In this paper, we propose a detection model named MAE-YOLO, providing an innovative solution for small object recognition problems in industrial inspection tasks for quadruped inspection robots. Through the MSESTE, ELCIN, and AMCFN modules, we significantly enhanced the model’s detection accuracy, robustness, and real-time performance in complex dynamic environments. Experimental results show that the MAE-YOLO model outperforms existing mainstream methods in small object recognition tasks. This research not only advances the application of quadruped robots in industrial automation and inspection but also offers new perspectives for computer vision technology in related fields.

Real-world deployment and testing on a quadruped robot platform is a critical and immediate next step. Future research may further optimize the lightweight design of the algorithm, enhancing robustness in resource-constrained environments, thereby supporting a wider range of industrial application scenarios.

## Data Availability

The datasets and code generated during this study are available in the following repositories: —VisDrone2019 Dataset: https://github.com/VisDrone/VisDrone-Dataset —MAE-YOLO Implementation: https://github.com/970334745/MAE-YOLO -Self-collected dataset: https://pan.baidu.com/s/1wke6gmmqdTYw3fziMkCevg?pwd=h38c
